# Melatonin, menopause, and thyroid function in gynecologic endocrinology: what is the role?

**DOI:** 10.1590/1806-9282.701EDIT

**Published:** 2024-03-15

**Affiliations:** 1 Universidade de São Paulo, Faculdade de Medicina, Hospital das Clínicas, Departamento de Obstetrícia e Ginecologia, Disciplina de Ginecologia, Laboratório de Ginecologia Estrutural e Molecular, São Paulo (SP), Brazil.; 2 General Hospital Novi Pazar, Department of Surgery – Novi Pazar, Serbia.; 3 Giresun University, Faculty of Medicine, Division of Endocrine Surgery – Giresun, Turkey.; 4 Giresun University, Faculty of Medicine, Department of General Surgery – Giresun, Turkey.; 5 University Clinical Center of Serbia, Department of Gynecology and Obstetrics, Clinic for Gynecology and Obstetrics – Belgrade, Serbia.; 6 Giresun University, Faculty of Medicine, Department of Pathology – Giresun, Turkey.; 7 General Hospital Novi Pazar, Department of Ophthalmology – Novi Pazar, Serbia.

Melatonin (MTN), a neurohormone primarily synthesized and secreted mainly by the pine cone-shaped gland of the cerebrum, named as the conarium or epiphysis cerebri, from amino acid tryptophan, was first isolated from the bovine pineal gland by Lerner et al^1^. However, the 17th-century philosopher Reneé Descartes hypothesized that the pineal gland of the brain, which remains poorly understood to date, represents the location of the *homo sapiens* soul; paleontologists described it as an ancestral "third eye"; and modern psychology declares perception beyond physical visual function^2,3^. This chemical messenger, *per se*, ensures high precision in the reconnoitering of the night period, is an endocrine marker for darkness, and participates in the regulation of circadian rhythm and the sleep-wake cycle. Of note, MTN can be produced by other organs, such as the brain, lungs, gastrointestinal tract, liver, thyroid, and reproductive and immune systems, and is present in mucus, saliva, breast milk, urine, sperm, amniotic fluid, Graafian follicle, etc.^1,4-9^. As such, various studies have shown that MTN affects many functions in the body and acts on different tissues, and some of its properties include significant antioxidant, anti-inflammatory, antiproliferative, and immunomodulatory capacity. MTN, *per se*, can directly neutralize toxic free radicals more effectively, suppresses chronic oxidative stress, has a significant impact on reproductive cells, enhances the quality of sperm and oocytes, has oncostatic and antitumoral cytoprotective effects^10,11^, alleviates some of the undesirable toxic effects of radiotherapy and chemotherapy by increasing the tolerance of healthy tissues, compared to other antioxidants, by stimulating responses to DNA damage. To this end, MTN's significant role in some chronic diseases, such as diabetes, blood glucose level regulation, and hypertension, has also been reported. MTN has been studied as a therapeutic option for many autoimmune diseases of multiple sclerosis, rheumatoid arthritis, and diabetes mellitus, based on its immunoregulatory properties. MTN achieves its effect through its receptors type 1 and type 2 (MT1 [Mel1^a^] and MT2 [Mel^1b^]) membrane-bound receptors. Moreover, the presence of the MT1 receptor in the thyroid gland has been proven, which indicates the possibility of MTN's influence on thyroid activity and hormone production. A third membrane-bound MTN-binding site, the MT3 receptor, was theorized as a biological target of MTN and was found to, in fact, act as the cytosolic enzyme, quinone reductase II (NQO2)^12^. Some authors have reported in their genetic study that the single-nucleotide polymorphism of MTN receptor type 1A, MTNR1A, coding the MT1 protein, was associated with a sensitivity to Graves’ disease and thyroid autoantibody formation, which supports the impression, that MTN can influence the development of autoimmune thyroid disease in thyroidology^13,14^. The thyroid gland is characterized by a high level of oxidative stress, and the use of pro-oxidants can lead to miscellaneous damage and diseases of this delicate papillon gland. Excessive iodine load, as an exogenous pro-oxidant, can induce apoptosis in the thyroid gland follicular cells. Furthermore, iodine compounds used in iodine prophylaxis also have potentially harmful effects. The thyroid gland is less sensitive to the pro-oxidative effects of potassium iodate, KIO^3^, and reacts more strongly to the antioxidant effect of MTN than other tissues, which plays a significant role in this phenomenon. Hence, MTN should be considered to avoid the potential damaging effects of iodine compounds applied in iodine prophylaxis^15-19^. Radiotherapy for head and neck tumors can often damage the thyroid follicular structure even though it is not affected by the tumor. However, administration of MTN before radiotherapy might attenuate the degree of tissue damage. Some authors pointed out the radioprotective effects of MTN in the acute phase of thyroid tissue damage. Besides the aforementioned characteristics, MTN has a crucial role in regulating human reproduction processes, such as oocyte quality, folliculogenesis, oocyte maturation, embryo implantation, fetal development, and the outcomes of pregnancy. Therefore, the idea of MTN utilization in the therapeutic approaches of reproductive and gestational disorders seems favorable to some authorities^20,21^. The low MTN levels in elderly people are correlated with reproductive aging and high gonadotropin secretion, while menopause is characterized by the inability of the ovaries to produce viable follicles and hormonal changes, which leads to menstrual cycle failures. Ovarian aging is characterized by reduced follicular reserve and augmented gonadotropin secretion^22^. In menopause, various alterations emerge in a woman's body, which results in the changes in her mental and physical health statuses. Anecdotally, women experience immuno-metabolic fluctuations, such as hormonal perturbations, sleep problems, and vasomotor symptoms, during menopause. Since MTN is involved in all these processes, it has the potential as a medication with multiple health benefits for the management of a menopausal woman^23,24^. In addition, some authors stated that MTN usage can improve physical symptoms such as sleep quality, mood state, estradiol levels, and body mass index in a menopausal woman, but not in the general menopausal ones^22^. In summary, MTN appears to play a key role in the regulation of the endocrine system, involving the regulation of gonadotrophin-releasing hormone (GnRH), promotion of progesterone synthesis, stimulation of oxytocin secretion, regulation of cortisol production, and promotion of androgen generation^12^. Nevertheless, it is critical to distinguish between the physiological effects and the pharmacological consequences of MTN administration due to specific differences in the response of any tissue to the agent and to determine whether the stage of development, sex differences, and genetic variability can affect how reproductive tissue responds to MTN by gynecologic endocrinologists and thyroidologists. As a matter of fact, this issue merits further investigation.
